# Supplementary material for: What do adolescents think about vaccines? Systematic review of qualitative studies
Source: PLOS Glob Public Health. 2022 Sep 29;2(9):e0001109. doi: 10.1371/journal.pgph.0001109 (PMC10022047; doi:10.1371/journal.pgph.0001109)
Supplement: S1 Text — (DOCX) [file pgph.0001109.s002.docx]

**SYSTEMATIC REVIEW WORKING TITLE:**

What do teenagers think about vaccines? Systematic review of qualitative studies.

START DATE: July 8, 2021

END DATE: September 12, 2021

**REVIEW TEAM MEMBERS:**

Hana Mitchell^1^, Rebecca Lim^2^, Prubjot Gill^3^ , Mohammed Al-Musawi ^4^ , Joban Dhanoa^5^, Julie Bettinger^6^

AFFILIATIONS

^1,2,4,5,6^ Vaccine Evaluation Center, BCCHR, University of British Columbia

^3^ UBC Library, University of British Columbia

**FUNDING:** Canadian Immunization Research Network

**BACKGROUND**

Several routine vaccines are approved and recommended during adolescence, including human papilloma virus (HPV) series, meningococcal vaccine, hepatitis B, and tetanus-diphtheria-acellular pertussis (Tdap) booster and most recently COVID-19. Adolescence presents a key opportunity to promote healthy lifestyle behaviors including vaccination, thereby positively affecting future health choices. While many adolescents do not independently decide about or consent to receive vaccines, some jurisdictions enable them to accept or refuse vaccination under a “mature minor” consent doctrine. The importance of understanding adolescents’ vaccine information needs and including them in vaccine decision making is increasingly recognized. Systematically summarizing data obtained from qualitative studies is therefore useful to inform and guide development of appropriate and acceptable healthcare services for particular populations and to identify knowledge gaps.

**RESEARCH AIM/ QUESTION**

Summarize the existing evidence on adolescent understanding of vaccines and their experiences with vaccine decision making

SUB-QUESTIONS:

1) What is adolescents' understanding of vaccines and vaccine preventable diseases?

2) What concerns do adolescents have about vaccines?

3) How do adolescents access, evaluate and utilize information about vaccines?

4) What is adolescents' experience with decision making about vaccines?

**STUDY POPULATION**: Adolescents age 10-19 (WHO definition of adolescence)

**INCLUSION CRITERIA**

- Full study text available in English
- Qualitative or mixed-methods study
- Qualitative data is described in sufficient detail to provide contextual information and to support study results (as evaluated by systematic review authors)
- Study includes participants age 10 to 19. Studies that also included data outside of this age group will be included provided the qualitative data from adolescents is reported separately
- Study reports primary data that is obtained directly from adolescents
- Study focuses on adolescents’ self-reported understanding of vaccines. Depending on the study and specific questions asked by study researchers understanding of vaccines could include a combination of adolescents describing their knowledge of vaccines and vaccine preventable diseases, their questions and concerns about vaccines, personal views and attitudes towards vaccines, as well as experiences with being involved in vaccine decision making.

**EXCLUSION CRITERIA**

- Full text not available in English
- Qualitative data obtained from adolescents is too thin to be suitable for analysis (as evaluated by systematic review authors with agreement reached by consensus)
- Study obtained qualitative data but reported it only quantitatively
- Non-primary data (i.e. policy briefs, opinions, reviews)
- Qualitative study focusing strictly on evaluating specific educational interventions (e.g. evaluation of a video game about vaccines) or experiences with injection (e.g. managing pain related to vaccination)
- Study only provides information about adolescents’ understanding of vaccines as described by other participants (e.g. teachers, parents or caretakers, healthcare providers), rather than adolescents themselves.

**SEARCHES:**

MEDLINE, Embase, CINAHL, PsycINFO

English language publications only

From inception until July 8, 2021

Search strategies are provided in the appendix

**DATA EXTRACTION/CODING**

Study title and abstract screening and full text screening will be done in Covidence with two out of three reviewers independently assessing each study, using predetermined inclusion and exclusion criteria. Disagreements will be resolved by consensus.

Relevant data capturing study characteristics and qualitative content will be extracted and entered into an Excel sheet in duplicate independently by two reviewers and then accuracy-checked and consolidated by a third reviewer. Disagreements will be resolved by consensus.

**QUALITY ASSESSMENT**

Quality will be assessed using Critical Appraisal Skills Program (©CASP) quality assessment tool independently by two reviewers.

**DATA SYNTHESIS**

Narrative summary of qualitative study findings and systematic level analysis will prepared by lead reviewer (HM) with input from all other reviewers. No meta-analysis/ meta-narrative will be undertaken.

**RESULTS SHARING**

Peer reviewed journal with focus on vaccinology and public health
