# Supplementary material for: What do adolescents think about vaccines? Systematic review of qualitative studies
Source: PLOS Glob Public Health. 2022 Sep 29;2(9):e0001109. doi: 10.1371/journal.pgph.0001109 (PMC10022047; doi:10.1371/journal.pgph.0001109)
Supplement: S1 Table — (DOCX) [file pgph.0001109.s003.docx]

S1 Table: SEARCH STRATEGIES

**MEDLINE (OVID)**

| **#** | **Query** |
| --- | --- |
| 1 | attitude to health/ |
| 2 | health knowledge, attitudes, practice/ |
| 3 | "patient acceptance of health care"/ |
| 4 | decision making/ |
| 5 | exp Informed Consent/ |
| 6 | (perception* or perceiv* or attitude* or knowledge or hesitan* or acceptance or acceptability or belief* or barrier* or facilitator* or self-consent or decision making or autonomy or information need* or view* or rejection or refusal or aware*).tw,kf. |
| 7 | or/1-6 |
| 8 | Child/ |
| 9 | (minors or grade 5 or grade five or grade 6 or grade six or grade 7 or grade seven or 10 year* old* or 11 year* old* or 12 year* old*).tw,kf. |
| 10 | (boy? or girl? or child* or juvenil* or kid? or paediatric* or pediatric* or minor or minors or school* or elementary student? or underage? or under-age?).tw,kf. |
| 11 | or/8-10 |
| 12 | Adolescent/ |
| 13 | (adolescent* or teenager* or teen* or youth*).tw,kf. |
| 14 | (grade 8 or grade eight or grade 9 or grade nine or grade 10 or grade ten or grade 11 or grade eleven or grade 12 or grade twelve or 13 year* old* or 14 year* old* or 15 year* old* or 16 year* old* or 17 year* old* or 18 year* old* or 19 year* old*).tw,kf. |
| 15 | or/12-14 |
| 16 | 11 or 15 |
| 17 | 7 and 16 |
| 18 | Vaccination/ |
| 19 | Mass Vaccination/ |
| 20 | Vaccination Refusal/ |
| 21 | Immunization Programs/ |
| 22 | or/18-21 |
| 23 | Vaccines/ |
| 24 | meningococcal vaccines/ or pertussis vaccine/ or diphtheria-tetanus-acellular pertussis vaccines/ or diphtheria-tetanus-pertussis vaccine/ or tetanus toxoid/ or diphtheria-tetanus vaccine/ or influenza vaccines/ or papillomavirus vaccines/ or human papillomavirus recombinant vaccine quadrivalent, types 6, 11, 16, 18/ or hepatitis b vaccines/ or COVID-19 Vaccines/ |
| 25 | 23 or 24 |
| 26 | (vaccin* or revaccinat* or immuniz* or immunis*).tw,kf. |
| 27 | ((meningococcal or pertussis or dtap or diphtheria or tetanus or tetanus toxoid or influenza or flu or laiv or papillomavirus or human papillomavirus recombinant or papilloma virus or HPV or hepatitis b or coronavirus or covid or cov or ncov or 2019-ncov or SARS-cov or SARS2 or SARS-CoV-2) adj2 vaccin*).tw,kf. |
| 28 | (menactra or menomune or menveo or gardasil or tetanus toxoid).tw,kf. |
| 29 | or/26-28 |
| 30 | 22 or 25 or 29 |
| 31 | 17 and 30 |
| 32 | ((("semi-structured" or semistructured or unstructured or informal or "in-depth" or indepth or "face-to-face" or structured or guide) adj2 (interview* or discussion* or questionnaire*)) or (focus group* or qualitative or ethnograph* or fieldwork or "field work" or "key informant")).tw,kw. or interviews as topic/ or focus groups/ or narration/ or qualitative research/ |
| 33 | 31 and 32 |

**EMBASE (OVID)**

| **#** | **Query** |
| --- | --- |
| 1 | attitude to health/ |
| 2 | patient attitude/ or patient attendance/ or patient compliance/ or patient dropout/ or patient participation/ or patient preference/ or patient satisfaction/ or refusal to participate/ or treatment refusal/ or vaccine hesitancy/ |
| 3 | decision making/ or ethical decision making/ or family decision making/ or medical decision making/ or patient decision making/ or shared decision making/ |
| 4 | informed consent/ |
| 5 | (perception* or perceiv* or attitude* or knowledge or hesitan* or acceptance or acceptability or belief* or barrier* or facilitator* or self-consent or decision making or autonomy or information need* or view* or rejection or refusal or aware*).tw,kw. |
| 6 | or/1-5 |
| 7 | child/ |
| 8 | (minors or grade 5 or grade five or grade 6 or grade six or grade 7 or grade seven or 10 year* old* or 11 year* old* or 12 year* old*).tw,kw. |
| 9 | (boy? or girl? or child* or juvenil* or kid? or paediatric* or pediatric* or minor or minors or school* or elementary student? or underage? or under-age?).tw,kw. |
| 10 | or/7-9 |
| 11 | adolescent/ |
| 12 | (adolescent* or teenager* or teen* or youth*).tw,kw. |
| 13 | (grade 8 or grade eight or grade 9 or grade nine or grade 10 or grade ten or grade 11 or grade eleven or grade 12 or grade twelve or 13 year* old* or 14 year* old* or 15 year* old* or 16 year* old* or 17 year* old* or 18 year* old* or 19 year* old*).tw,kw. |
| 14 | or/11-13 |
| 15 | 10 or 14 |
| 16 | 6 and 15 |
| 17 | mass immunization/ or vaccination/ or vaccination coverage/ |
| 18 | vaccination refusal/ |
| 19 | vaccine/ |
| 20 | Meningococcus vaccine/ or pertussis vaccine/ or diphtheria tetanus pertussis vaccine/ or tetanus toxoid/ or influenza vaccine/ or Wart virus vaccine/ or hepatitis B vaccine/ or SARS-CoV-2 vaccine/ |
| 21 | (vaccin* or revaccinat* or immuniz* or immunis*).tw,kw. |
| 22 | ((meningococcal or pertussis or dtap or diphtheria or tetanus or tetanus toxoid or influenza or flu or laiv or papillomavirus or human papillomavirus recombinant or papilloma virus or HPV or hepatitis b or coronavirus or covid or cov or ncov or 2019-ncov or SARS-cov or SARS2 or SARS-CoV-2) adj2 vaccin*).tw,kw. |
| 23 | (menactra or menomune or menveo or gardasil or tetanus toxoid).tw,kw. |
| 24 | or/17-23 |
| 25 | 16 and 24 |
| 26 | (("semi-structured" or semistructured or unstructured or informal or "in-depth" or indepth or "face-to-face" or structured or guide) adj3 (interview* or discussion* or questionnaire*)).ti,ab. or (focus group* or qualitative or ethnograph* or fieldwork or "field work" or "key informant").tw,kw. or qualitative research/ |
| 27 | 25 and 26 |

**PSYCHINFO (EBSCO)**

| **#** | **Query** |
| --- | --- |
| S22 | S20 AND S21 |
| S21 | ((TI ( experiences or interview* or qualitative ) OR AB ( experiences or interview* or qualitative )) |
| S20 | S14 AND S19 |
| S19 | S15 OR S16 OR S17 OR S18 |
| S18 | TI ( (menactra or menomune or menveo or gardasil or tetanus toxoid) ) OR AB ( (menactra or menomune or menveo or gardasil or tetanus toxoid) ) OR KW ( (menactra or menomune or menveo or gardasil or tetanus toxoid) ) |
| S17 | TI ( ((meningococcal or pertussis or dtap or diphtheria or tetanus or tetanus toxoid or influenza or flu or laiv or papillomavirus or human papillomavirus recombinant or papilloma virus or HPV or hepatitis b or coronavirus or covid or cov or ncov or 2019-ncov or SARS-cov or SARS2 or SARS-CoV-2) N2 vaccin*) ) OR AB ( ((meningococcal or pertussis or dtap or diphtheria or tetanus or tetanus toxoid or influenza or flu or laiv or papillomavirus or human papillomavirus recombinant or papilloma virus or HPV or hepatitis b or coronavirus or covid or cov or ncov or 2019-ncov or SARS-cov or SARS2 or SARS-CoV-2) N2 vaccin*) ) OR KW ( ((meningococcal or pertussis or dtap or diphtheria or tetanus or tetanus toxoid or influenza or flu or laiv or papillomavirus or human papillomavirus recombinant or papilloma virus or HPV or hepatitis b or coronavirus or covid or cov or ncov or 2019-ncov or SARS-cov or SARS2 or SARS-CoV-2) N2 vaccin*) ) |
| S16 | TI ( (vaccin* or revaccinat* or immuniz* or immunis*) ) OR AB ( (vaccin* or revaccinat* or immuniz* or immunis*) ) OR KW ( (vaccin* or revaccinat* or immuniz* or immunis*) ) |
| S15 | DE "Immunization" |
| S14 | (S12 AND S13) |
| S13 | S6 OR S7 OR S8 OR S9 OR S10 OR S11 |
| S12 | S1 OR S2 OR S3 OR S4 OR S5 |
| S11 | ( TI ( (grade 8 or grade eight or grade 9 or grade nine or grade 10 or grade ten or grade 11 or grade eleven or grade 12 or grade twelve or 13 year* old* or 14 year* old* or 15 year* old* or 16 year* old* or 17 year* old* or 18 year* old* or 19 year* old*) ) OR AB ( (grade 8 or grade eight or grade 9 or grade nine or grade 10 or grade ten or grade 11 or grade eleven or grade 12 or grade twelve or 13 year* old* or 14 year* old* or 15 year* old* or 16 year* old* or 17 year* old* or 18 year* old* or 19 year* old*) ) ) OR KW ( ( (grade 8 or grade eight or grade 9 or grade nine or grade 10 or grade ten or grade 11 or grade eleven or grade 12 or grade twelve or 13 year* old* or 14 year* old* or 15 year* old* or 16 year* old* or 17 year* old* or 18 year* old* or 19 year* old*) ) ) |
| S10 | ( TI ( (adolescent* or teenager* or teen* or youth*) ) OR AB ( (adolescent* or teenager* or teen* or youth*) ) ) OR KW ( ( (adolescent* or teenager* or teen* or youth*) ) ) |
| S9 | DE "Adolescent Health" OR DE "Adolescent Characteristics" OR DE "Adolescent Attitudes" OR DE "Early Adolescence" OR DE "Adolescent Behavior" |
| S8 | ( TI ( (boy? or girl? or child* or juvenil* or kid? or paediatric* or pediatric* or minor or minors or school* or elementary student? or underage? or under-age?) ) OR AB ( (boy? or girl? or child* or juvenil* or kid? or paediatric* or pediatric* or minor or minors or school* or elementary student? or underage? or under-age?) ) ) OR KW ( ( (boy? or girl? or child* or juvenil* or kid? or paediatric* or pediatric* or minor or minors or school* or elementary student? or underage? or under-age?) ) ) |
| S7 | ( TI ( (minors or grade 5 or grade five or grade 6 or grade six or grade 7 or grade seven or 10 year* old* or 11 year* old* or 12 year* old*) ) OR AB ( (minors or grade 5 or grade five or grade 6 or grade six or grade 7 or grade seven or 10 year* old* or 11 year* old* or 12 year* old*) ) ) OR KW ( ( (minors or grade 5 or grade five or grade 6 or grade six or grade 7 or grade seven or 10 year* old* or 11 year* old* or 12 year* old*) ) ) |
| S6 | DE "Child Health" OR DE "Child Attitudes" |
| S5 | ( TI ( (perception* or perceiv* or attitude* or knowledge or hesitan* or acceptance or acceptability or belief* or barrier* or facilitator* or self-consent or decision making or autonomy or information need* or view* or rejection or refusal or aware*) ) OR AB ( (perception* or perceiv* or attitude* or knowledge or hesitan* or acceptance or acceptability or belief* or barrier* or facilitator* or self-consent or decision making or autonomy or information need* or view* or rejection or refusal or aware*) ) ) OR KW ( ( (perception* or perceiv* or attitude* or knowledge or hesitan* or acceptance or acceptability or belief* or barrier* or facilitator* or self-consent or decision making or autonomy or information need* or view* or rejection or refusal or aware*) ) ) |
| S4 | DE "Informed Consent" |
| S3 | DE "Decision Making" |
| S2 | DE "Health Knowledge" |
| S1 | DE "Public Health Attitudes" OR DE "Health Attitudes" OR DE "Health Literacy" |

| CINAHL (EBSCO)  \| \| **#** \| \| **Query** \| \| --- \| --- \| --- \| \| S23 \| S21 AND S22 \| \| \| S22 \| TI interview OR AB interview OR (MH "Audiorecording") OR AB "qualitative stud*" OR (MH "Qualitative Stud*") OR TI "qualitative stud*" \| \| \| S21 \| S15 AND S20 \| \| \| S20 \| S16 OR S17 OR S18 OR S19 \| \| \| S19 \| TI ( menactra or menomune or menveo or gardasil or tetanus toxoid ) OR AB ( menactra or menomune or menveo or gardasil or tetanus toxoid ) \| \| \| S18 \| TI ( meningococcal or pertussis or dtap or diphtheria or tetanus or tetanus toxoid or influenza or flu or laiv or papillomavirus or human papillomavirus recombinant or papilloma virus or HPV or hepatitis or coronavirus or covid or SARS-CoV-2) N2 vaccin* ) OR AB ( meningococcal or pertussis or dtap or diphtheria or tetanus or tetanus toxoid or influenza or flu or laiv or papillomavirus or human papillomavirus recombinant or papilloma virus or HPV or hepatitis or coronavirus or covid or SARS-CoV-2) N2 vaccin*) \| \| \| S17 \| (MH "Vaccines+") OR (MH "Bacterial Vaccines+") OR (MH "Cancer Vaccines+") OR (MH "Toxoids+") OR (MH "Vaccines, Combined+") OR (MH "Viral Vaccines+") \| \| \| S16 \| (MH "Immunization") OR (MH "Anti-Vaccination Movement") OR (MH "Vaccination Coverage") \| \| \| S15 \| S5 AND S14 \| \| \| S14 \| S9 OR S13 \| \| \| S13 \| S10 OR S11 OR S12 \| \| \| S12 \| TI (grade 8 or grade eight or grade 9 or grade nine or grade 10 or grade ten or grade 11 or grade eleven or grade 12 or grade twelve or 13 year* old* or 14 year* old* or 15 year* old* or 16 year* old* or 17 year* old* or 18 year* old* or 19 year* old*) OR AB (grade 8 or grade eight or grade 9 or grade nine or grade 10 or grade ten or grade 11 or grade eleven or grade 12 or grade twelve or 13 year* old* or 14 year* old* or 15 year* old* or 16 year* old* or 17 year* old* or 18 year* old* or 19 year* old*) \| \| \| S11 \| TI (adolescent* or teenager* or teen* or youth*) OR AB (adolescent* or teenager* or teen* or youth*) \| \| \| S10 \| (MH "Adolescence") OR (MH "Adolescent Health") \| \| \| S9 \| S6 OR S7 OR S8 \| \| \| S8 \| TI ( boy? or girl? or child* or juvenil* or kid? or paediatric* or pediatric* or minor or minors or school* or elementary student? or underage? or under-age? ) OR AB ( boy? or girl? or child* or juvenil* or kid? or paediatric* or pediatric* or minor or minors or school* or elementary student? or underage? or under-age? ) \| \| \| S7 \| TI ( minors or grade 5 or grade five or grade 6 or grade six or grade 7 or grade seven or 10 year* old* or 11 year* old* or 12 year* old* ) OR AB ( minors or grade 5 or grade five or grade 6 or grade six or grade 7 or grade seven or 10 year* old* or 11 year* old* or 12 year* old* ) \| \| \| S6 \| (MH "Child") OR (MH "Child Health") \| \| \| S5 \| S1 OR S2 OR S3 OR S4 \| \| \| S4 \| TI (perception* or perceiv* or attitude* or knowledge or hesitan* or acceptance or acceptability or belief* or barrier* or facilitator* or self-consent or decision making or autonomy or information need* or view* or rejection or refusal or aware*) OR AB (perception* or perceiv* or attitude* or knowledge or hesitan* or acceptance or acceptability or belief* or barrier* or facilitator* or self-consent or decision making or autonomy or information need* or view* or rejection or refusal or aware*) \| \| \| S3 \| (MH "Consent+") \| \| \| S2 \| (MH "Decision Making+") OR (MH "Decision Making, Ethical") OR (MH "Decision Making, Family") OR (MH "Decision Making, Shared") OR (MH "Decision Making, Patient+") \| \| \| S1 \| (MH "Attitude to Health+") OR (MH "Health Beliefs") OR (MH "Patient Compliance+") OR (MH "Patient Satisfaction+") OR (MH "Patient Preference") \| \| \| \| --- \| --- \| --- \| --- \| --- \| --- \| --- \| --- \| --- \| --- \| --- \| --- \| --- \| --- \| --- \| --- \| --- \| --- \| --- \| --- \| --- \| --- \| --- \| --- \| --- \| --- \| --- \| --- \| --- \| --- \| --- \| --- \| --- \| --- \| --- \| --- \| --- \| --- \| --- \| --- \| --- \| --- \| --- \| --- \| --- \| --- \| --- \| --- \| --- \| --- \| --- \| --- \| --- \| --- \| --- \| --- \| --- \| --- \| --- \| --- \| --- \| --- \| --- \| --- \| --- \| --- \| --- \| --- \| --- \| --- \| --- \| --- \| --- \| |
| --- | --- | --- | --- | --- | --- | --- | --- | --- | --- | --- | --- | --- | --- | --- | --- | --- | --- | --- | --- | --- | --- | --- | --- | --- | --- | --- | --- | --- | --- | --- | --- | --- | --- | --- | --- | --- | --- | --- | --- | --- | --- | --- | --- | --- | --- | --- | --- | --- | --- | --- | --- | --- | --- | --- | --- | --- | --- | --- | --- | --- | --- | --- | --- | --- | --- | --- | --- | --- | --- | --- | --- | --- | --- |
|  |
