# Supplementary material for: What do adolescents think about vaccines? Systematic review of qualitative studies
Source: PLOS Glob Public Health. 2022 Sep 29;2(9):e0001109. doi: 10.1371/journal.pgph.0001109 (PMC10022047; doi:10.1371/journal.pgph.0001109)
Supplement: Attachment — Submitted filename: CIRN27-321 Response to reviewers.docx [file pgph.0001109.s004.docx]

Thank you for the thoughtful review. We have responded to reviewer one’s suggestions below.

Abstract
- Do you mean high-income settings (in any country) or high-income countries? If the latter, it would provide clarity to use the word countries instead of settings.

We were referring to high-income countries and have changed the terminology accordingly.

- Would “perspectives from sexual and gender minorities” or something be a better term than “homosexual and non-binary adolescents”? The latter does not include e.g. transgender adolescents who may have very specific needs or concerns around certain vaccines. It looks to me like the APA guidelines on inclusive language are quite helpful: <https://www.apa.org/about/apa/equity-diversity-inclusion/language-guidelines> (includes links to even more specific guides on sexual orientation and gender if relevant, please also note the caveats on the term “minorities”)

Thanks for this suggestion. We have made this change.

- How was the evidence synthesised? Some brief details about methods needed in abstract.

This has been added to the abstract and methods section.

- If there is space, I think it could be useful to expand “media” to “traditional and online media” (in line with your discussion) or something else more specific as readers may assume media to only mean traditional mass media, whereas social media would be expected to be particularly relevant for adolescents.

Done.

Methods
- I am impressed that each record seems to have been double-screened, quite an undertaking!

Thanks!

- This section needs more details about the narrative synthesis approach and process (e.g. what data were extracted and how, how were the sub-headings of the results section developed – in advance or more inductively during the synthesis?)

This has been added, along with references justifying methodological approach.

Results
- I do not think “gender orientation” is typically used – for gender, the recommended terms would be “gender identity” or “gender expression” (depending on exact context), and orientation is used in the term “sexual orientation”.

This has been revised to gender identity.

- Table 1 is very useful! Just check spelling, e.g. “lead” should be “led”.

Done.

- I am very glad to see how comprehensively the individual studies are indicated in the text – it really helps to trace the evidence in the synthesis (commenting on this because not all reviews do this but it is so valuable and much more transparent!).

Thanks.

- For clarity, “vaccine handouts” may be better described as “vaccine information sheets” or similar (handout is used at least twice and may confuse reader to think it is about cost, not information).

Revised.

- The word “vaccine” is missing when referring to the HPV vaccine on line 282 (starts with Sweden) and 283.

Revised, thanks for catching this typo.

Discussion
- Generally very comprehensive and interesting!
- Elaborate on why inclusion and consideration of sexual and gender minorities is so important based on the sources you cite – I agree that this is key but it may not be fully obvious to every reader and the relevance of course varies somewhat depending on the vaccine and the programme/policy (e.g. HPV vaccine programmes only targeting girls would have implications for inclusion of transgender and non-binary adolescents).

We have added additional discussion on this.

Conclusion
- Again, I would recommend using “sexual and gender minorities” or at least avoiding “gender orientation” and using “identity” instead (but I think you mean both gender and sexuality here so relevant to mention both).

Journal Requirements:

1. Please amend your detailed online Financial Disclosure statement. This is published with the article. It must therefore be completed in full sentences and contain the exact wording you wish to be published.

Done.

State the initials, alongside each funding source, of each author to receive each grant.

Done.

2. Please ensure that the funders and grant numbers match between the Financial Disclosure field and the Funding Information tab in your submission form. Note that the funders must be provided in the same order in both places as well.

Done.

3. Please update your online Competing Interests statement. If you have no competing interests to declare, please state: “The authors have declared that no competing interests exist.”

Done.

4. Please provide separate figure file in .tif or .eps format and ensure that all files are under our size limit of 10MB.

Done.

5. Please ensure that you refer to Table 1 in your text as, if accepted, production will need this reference to link the reader to the table.

Done.

6. We have noticed that you have uploaded Supporting Information files, but you have not included a list of legends. Please add a full list of legends for your Supporting Information files after the references list.

This has been added.

7. Please review your reference list to ensure that it is complete and correct. If you have cited papers that have been retracted, please include the rationale for doing so in the manuscript text, or remove these references and replace them with relevant current references. Any changes to the reference list should be mentioned in the rebuttal letter that accompanies your revised manuscript. If you need to cite a retracted article, indicate the article’s retracted status in the References list and also include a citation and full reference for the retraction notice.

We have reviewed the reference list and confirm that it is complete and correct. To our best knowledge none of the papers cited have been retracted.

The following references have been added to provide additional context, based on reviewers’ comments.

26. Lucas PJ, Baird J, Arai L, Law C, Roberts HM. Worked examples of alternative methods for the synthesis of qualitative and quantitative research in systematic reviews. BMC Med Res Methodol. 2007 Jan 15;7:4.

27.	Barnett-Page E, Thomas J. Methods for the synthesis of qualitative research: a critical review. BMC Med Res Methodol. 2009 Aug 11;9:59.

112.	Pho AT, Mangal S, Bakken S. Human Papillomavirus Vaccination Among Transgender and Gender Diverse People in the United States: An Integrative Review. Transgender Health. 2022 Aug;7(4):303–13.

113.	Bednarczyk RA, Whitehead JL, Stephenson R. Moving beyond sex: Assessing the impact of gender identity on human papillomavirus vaccine recommendations and uptake among a national sample of rural-residing LGBT young adults. Papillomavirus Res. 2017 Apr 13;3:121–5.

114.	Heidari S, Babor TF, De Castro P, Tort S, Curno M. Sex and Gender Equity in Research: rationale for the SAGER guidelines and recommended use. Res Integr Peer Rev. 2016 May 3;1(1):2.
