# Supplementary material for: What do adolescents think about vaccines? Systematic review of qualitative studies
Source: PLOS Glob Public Health. 2022 Sep 29;2(9):e0001109. doi: 10.1371/journal.pgph.0001109 (PMC10022047; doi:10.1371/journal.pgph.0001109)
Supplement: Attachment — Submitted filename: CIRN27-324 2nd response to reviewers.docx [file pgph.0001109.s005.docx]

1. Specifying that you are referring to "sexual orientation" - there are several instances where the word "orientation" is used alone.

Yes, we have added sexual to all instances where orientation is used.

2. Elaborating in the sentence "Only 4 studies specifically reported participants’ gender identity" that the point you are making is that only four studies reported how participants self-identify in terms of gender, or Your point is that only four included any information about participant gender.

It is the former, only four studies captured data on how participants self-identify in terms of gender. We have revised the wording to make this clear.

Journal Requirements:

1. We do not publish any copyright or trademark symbols that usually accompany proprietary names, eg (R), (C), or TM (e.g. next to drug or reagent names). Please remove all instances of trademark/copyright symbols throughout the text, including ©, ®, ™ on page 17.

Done.

2. Please review your reference list to ensure that it is complete and correct. If you have cited papers that have been retracted, please include the rationale for doing so in the manuscript text, or remove these references and replace them with relevant current references. Any changes to the reference list should be mentioned in the rebuttal letter that accompanies your revised manuscript. If you need to cite a retracted article, indicate the article’s retracted status in the References list and also include a citation and full reference for the retraction notice.

We have reviewed the references and they are correct to the best of our knowledge. We are not aware of any that were retracted. If we have missed this please let us know which reference and we will revise.
